# Identification of candidate genes for soybean seed coat-related traits using QTL mapping and GWAS

**DOI:** 10.3389/fpls.2023.1190503

**Published:** 2023-06-13

**Authors:** Yue Yang, Tiantian Zhao, Fengmin Wang, Luping Liu, Bingqiang Liu, Kai Zhang, Jun Qin, Chunyan Yang, Yake Qiao

**Affiliations:** ^1^ College of Agronomy and Biotechnology, Hebei Normal University of Science and Technology, Qinhuangdao, China; ^2^ Hebei Laboratory of Crop Genetics and Breeding, National Soybean Improvement Center Shijiazhuang Sub-Center, Huang-Huai-Hai Key Laboratory of Biology and Genetic Improvement of Soybean, Ministry of Agriculture and Rural Affairs, Institute of Cereal and Oil Crops, Hebei Academy of Agricultural and Forestry Sciences, Shijiazhuang, Hebei, China; ^3^ Hebei Key Laboratory of Molecular and Cellular Biology, Key Laboratory of Molecular and Cellular Biology of Ministry of Education, Hebei Collaboration Innovation Center for Cell Signaling, College of Life Science, Hebei Normal University, Shijiazhuang, China

**Keywords:** seed coat color, QTL, soybean, association analysis, glutathione-s-transferase

## Abstract

Seed coat color is a typical morphological trait that can be used to reveal the evolution of soybean. The study of seed coat color-related traits in soybeans is of great significance for both evolutionary theory and breeding practices. In this study, 180 F_10_ recombinant inbred lines (RILs) derived from the cross between the yellow-seed coat cultivar Jidou12 (ZDD23040, JD12) and the wild black-seed coat accession Y9 (ZYD02739) were used as materials. Three methods, single-marker analysis (SMA), interval mapping (IM), and inclusive composite interval mapping (ICIM), were used to identify quantitative trait loci (QTLs) controlling seed coat color and seed hilum color. Simultaneously, two genome-wide association study (GWAS) models, the generalized linear model (GLM) and mixed linear model (MLM), were used to jointly identify seed coat color and seed hilum color QTLs in 250 natural populations. By integrating the results from QTL mapping and GWAS analysis, we identified two stable QTLs (*qSCC02* and *qSCC08*) associated with seed coat color and one stable QTL (*qSHC08*) related to seed hilum color. By combining the results of linkage analysis and association analysis, two stable QTLs (*qSCC02*, *qSCC08*) for seed coat color and one stable QTL (*qSHC08*) for seed hilum color were identified. Upon further investigation using Kyoto Encyclopedia of Genes and Genomes (KEGG) analysis, we validated the previous findings that two candidate genes (*CHS3C* and *CHS4A*) reside within the *qSCC08* region and identified a new QTL, *qSCC02*. There were a total of 28 candidate genes in the interval, among which *Glyma.02G024600*, *Glyma.02G024700*, and *Glyma.02G024800* were mapped to the glutathione metabolic pathway, which is related to the transport or accumulation of anthocyanin. We considered the three genes as potential candidate genes for soybean seed coat-related traits. The QTLs and candidate genes detected in this study provide a foundation for further understanding the genetic mechanisms underlying soybean seed coat color and seed hilum color and are of significant value in marker-assisted breeding.

## Introduction

Soybean [*Glycine max* (L.) Merr.] is the most widely cultivated crop in the world. The color of the soybean seed coat is an important agronomic trait that determines seed quality and is also an evolutionary trait (Qiu, 2006; Qiu et al., 2021). Through domestication and natural evolution, soybean seed coat color has gradually evolved to green, brown, gray, and bicolor (Xu et al., 2013). Predominantly featuring yellow seed coats, contemporary soybean varieties exhibit color variations primarily at the hilum. Conversely, black seed coats and black hilum colors are typically displayed by their wild soybean counterparts. Depending on the depth of color, seed coats are further categorized into various types. For example, brown seed coats are divided into dark brown and light brown, and green-seed coat soybeans are commonly referred to as green beans (Wang et al., 2020) and further divided into dark green and light green. The formation of black or brown-colored seeds is primarily due to the accumulation of metabolic products such as flavonoids and anthocyanins within the epidermal layer of the seed coat (Sun et al., 2015). These bioactive compounds have been the focus of extensive research, as they possess remarkable antioxidant properties, which contribute to potential health benefits. Additionally, these compounds are known to influence the flavor profiles of the seeds, thus making them desirable for certain culinary applications (Dixon, 2003). Soybean seed coat color holds a strong connection to both the appearance and quality of the seeds. As such, investigating soybean seed coat coloration carries not only significant theoretical importance but also practical implications for the cultivation and utilization of soybeans (Song et al., 2012; Song et al., 2016).

The natural products causing differences in seed coat color and seed hilum color include flavonoids and anthocyanins (Zabala and Vodkin, 2003; Song et al., 2012). Flavonoids are a family of aromatic molecules derived from phenylalanine and acetyl-CoA through the fatty acid pathway. Primarily, the presence of two anthocyanidin glycosides (anthocyanins)-cyanidin-3-monoglucoside and delphinidin-3-monoglucoside – is responsible for the wild-type characteristic of black seed coats in soybeans (Yoshikura and Hamaguchi, 1969; Winkel-Shirley, 2001). Kovinich et al. (2012) reported that when the expression of both the *ANR1* (proanthocyanidin reductase 1) and *ANR2* (proanthocyanidin reductase 2) genes was inhibited, the seed coat of soybeans showed a distinctive red−brown color. The coloration of soybean seed coats is primarily governed by five classic genetic loci, which are designated as *I*, *R*, *T*, *W1*, and *O* (Palmer et al., 2004; Yang et al., 2010). The synthesis of seed coat pigments, which primarily determines seed coat color, is predominantly regulated by three independent loci, namely *I*, *R*, and *T* (Song et al., 2016). The *I* locus is located in the molecular linkage group A2 of soybean and contains the chalcone synthase (CHS) gene cluster (Todd, 1996; Clough et al., 2004; Tuteja, 2004). In lines possessing the recessive (*i*) allele, seed coats can be brown, partially black, buff, or black, contingent on the allelic state of the *Tawny R* and *W1* loci. The impact of the *O* and *W1* loci on seed coat color is exclusive to the homozygous recessive *ir* or *it* genotype, respectively (Palmer et al., 2004). Furthermore, In conjunction with genotypes at the *I*, *W1*, *R*, and *O* loci, the *T* locus generally contributes to the darkening of the hilum and/or seed coat color (Carpentieri-Pípolo et al., 2007; Guo and Qiu, 2013).

With the advancement of sequencing and molecular biology techniques, breeders and geneticists have been exploring quantitative trait loci (QTL) and candidate genes related to seed coat color and seed characteristics through positional studies. QTL mapping analysis is an important method for uncovering genes that control quantitative traits. To date, over 30 molecular markers controlling soybean seed coat color have been detected on different chromosomes. For example, Yu et al. (2021) used SLAF_seq combined with the bulk segregant analysis (BSA) method to locate the genes on chromosomes 5, 11, 12, 19, and 20 controlling the brown seed coat trait in soybeans. Dong et al. (2020) used simple sequence repeat (SSR) markers to study the yellow-seed coat soybean cultivar 09-95, its mutant brown-seed coat cultivar H09-95 and the brown-seed coat cultivar Beidou 14 in the F2 population. The study revealed that the mutated seed coat color gene was located between SAT-162 and SSR-53 in the A2 linkage group. Liu et al. (2021) used the SojacsSLP5 population of wild soybean improved by NN1138-2 (Max) × N24852 (Soja) as the material and combined high-density SNP LDB-MAP with RNA-Seq and DNA resequencing data from the parents to predict two candidate genes for seed coat color. Genome-wide association study (GWAS) is also a major method for mining genetic information about important traits in crops (Wan et al., 2019). In recent years, GWAS technology has been successfully applied to the genetic analysis of important traits in soybeans, and a series of research developments have been made (Wan et al., 2019). Song et al. (2020) identified four loci related to seed coat color through association and linkage analysis, located in the regions of BARCSOYSSR_1_1503 and 1_1546 on chromosome 01, 6-942 and 6_998 on chromosome 06, 8_459 and 8_480 on chromosome 08, and Sat_352 and Satt_196 on chromosome 09. Through a genome-wide association analysis of a natural population composed of three types of soybeans, namely, wild soybeans, semiwild soybeans and cultivated soybeans, Li (2021) detected a total of 182 and 130 significant loci when using generalized linear model (GLM) and mixed linear model (MLM), respectively. This analysis also revealed the presence of 24 genes that are associated with seed coat pigmentation.

Anthocyanins are thought to have diverse human health-promoting capabilities (Li et al., 2018). Despite previous research on soybean seed coat color, the genetic mechanism controlling seed coat color remains elusive, and there is a lack of research on the identification of seed coat-related candidate genes. In this study, distant wild soybean and cultivated soybean recombinant inbred lines (RILs) and natural populations with large genetic background differences are used to provide rich genetic research materials for further study of the genetic mechanism underlying seed coat variation. Specifically, we utilized 180 F_10_ RILs including cultivated soybeans and wild soybeans with large differences in genetic background and a population of 250 natural accessions as materials. Three models in IciMapping 4.2.53 software were used to detect seed coat color and hilum color-related loci in the RILs. Two types of models, the GLM and MLM, were used to perform a GWAS of the 250 natural accessions using TASSEL 5.0 software. By integrating linkage analysis and association analysis, this study aims to identify the QTLs related to seed coat traits of soybeans and predict the candidate genes that control seed coat-related traits, providing a research foundation for soybean breeding.

## Materials and methods

### Plant materials and field trials

The materials used in this study were a set of 180 RILs derived from the cross between Jidou12 (ZDD23040, JD12), a yellow-seeded and yellow-hilum soybean variety developed by the Hebei Academy of Agriculture and Forestry Sciences, and Ye 9 (ZYD02739, Y9), a wild soybean variety with black seed coats and a black hilum. The two parents different greatly in seed coat and hilum colors. A population of 250 natural accessions was provided by Dr. Lijuan Qiu’s from the Chinese Academy of Agricultural Sciences, including 60 accessions of the Chinese mini core collection, 108 typical varieties from China, and 82 foreign accessions from the United States, South Korea, and Japan. The parent plants, 180 RIL populations, and 250 accessions in the experimental design. The experiment was conducted in three separate replications during the years 2019, 2020, and 2021. Experiment was conducted at Dishang Experimental Farm (Longitude: 114.72° E, Latitude: 37.95° N) of the Institute of Cereal and Oil Crops, under the Hebei Academy of Agricultural and Forestry Sciences in Shijiazhuang City, China. In each replication, we employed a randomized complete block design (RCBD) to plant these materials. A total of 6750 rows were planted, with 2250 rows in each environment (year). Within each environment, each experimental material had three replicates. Each replicate included one plot, resulting in three plots for each experimental material in each environment. This ensured a high level of reliability and accuracy in the experimental design. The plant density used in the experiment was 12,000 plants per mu (approximately 1,974 plants per hectare). To better assess phenotypic differences, we randomly selected 10 plants from the middle row of each plot and evaluated their phenotypes at the R8 (full maturity) stage (Dornbos and McDonald, 1986).

### Trait evaluation

The color of soybean seeds was manually evaluated according to the “Soybean Phenotypic Descriptor and Data Standard” for both the RILs and natural population (Qiu, 2006), and the distributions of seed coat color and seed hilum color within each population were statistically analyzed (**Tables S6**, **S7**). The seed coat color were represented by numbers 1 to 10 for the RILs, indicating yellow, yellow−green, light green, green, dark green, yellow−brown, light brown, dark brown, brown, and black, respectively. For the seed coat color in 250 accessions, the colors were represented by numbers 1 to 7, indicating yellow, green, color-mixing, mixed-brown, brown, mixed-black, and black respectively, and by numbers 1 to 8 for the seed hilum color in 250 accessions, indicating yellow, light brown, light gray, dark brown, brown, gray, light black and black respectively. For the seed hilum color in RILs, the colors were represented by numbers 1 to 8, indicating light yellow, yellow, yellow-green, green, light brown, brown. dark brown and black. Phenotypic data were collected and analyzed using Microsoft Office Excel 11.1.0.10314 and SPSS 3.0 software, and graphs were generated by Graphpad Prism 8.0.

### GBS library construction and SNP identification

For both populations, the cetyltrimethylammonium bromide (CTAB) method was used to extract DNA from seedling leaves (Allen et al., 2006). For the RIL population, DNA samples were randomly sheared to an average length of approximately 350 bp using a Covaris sonicator. The library was constructed using the TruSeq Library Construction Kit (Novogene, Beijing, China) and sequenced on the Illumina HiSeq platform. The sequencing data were compared with the reference genome (G.max WM82. A2) using BWA software (Li and Durbin, 2009) with the following parameters: MEM-T4-K32-M. SAM tools software (Li et al., 2009) was used for format conversion and SNP detection, with a minimum SNP length of 4 bp and a minimum quality (MQ) value of 20. In comparison to the Williams 82.A1.V1 (https://www.soybase.org/GlycineBlastPages/archives/Gma1.01.20140304.fasta.zip), a total of 6288 high-quality SNPs were identified and retained for subsequent analysis (**Table S10**).

250 DNA samples were processed using the ApeKI restriction enzyme following the protocol established by Elshire et al. (2011). For each accession, the genotyping-by-sequencing (GBS) dataset comprised 3.26 million short reads, which accounted for 283.74 Mbp of sequences. Approximately half a million SNPs were identified by SNP calling. SNPs with a minor allele frequency (MAF) less than 5%, heterozygosity greater than 10% and/or over 15% missing data were eliminated. Compared with the genome of Williams 82.A1.V1 (https://www.soybase.org/GlycineBlastPages/archives/Gma1.01.20140304.fasta.zip), a total of 11,860 high-quality SNPs were retained and used for further analysis (**Table S9**).

### Bin map construction and QTL analysis

To minimize redundancy in markers, the GBS data was filtered based on segregation patterns observed in the RILs using the BIN function within IciMapping V4.2.53 (Li et al., 2008; Meng et al., 2015). Markers that displayed segregation with at least one other marker were retained, and a representative marker was chosen to represent each bin (Zeng et al., 2019). The selected markers were subsequently employed to construct the linkage map utilizing the MAP function in IciMapping (V4.2.53) software (https://www.isbreeding.net/software/). Following this, QTLs were detected using ICIM, SMA, and IM within the biparental population (BIP) model of QTL IciMapping software with a PIN of 0.01. A significance level of 0.05 was established using 1000 permutations to assess the statistical significance of QTL effects (Pei et al., 2018).

Three distinct methods were employed to identify quantitative trait loci (QTL): inclusive composite interval mapping (ICIM), single-marker analysis (SMA), and interval mapping (IM). These methods were applied within the biparental population (BIP) model of QTL IciMapping software (Version 4.2.53). A P value of 0.01 was set as the threshold for entering variables (PIN). To evaluate the statistical significance of QTL effects, a logarithm of the odds (LOD) score threshold was determined by conducting 1,000 permutations at a 0.05 significance level, following the approach outlined by Pei et al. (2018).

### Population structure analysis and GWAS

Population structure analysis (PCA) was conducted on 250 samples utilizing the Bayesian method in Structure 2.3.4. A total of 5129 loci were randomly chosen from 11,860 SNPs to infer population structure (K) using a mixed model based on allele frequencies. To achieve an even distribution of SNPs across chromosomes, we employed a Python (version 3.8.8) script specifically designed for this purpose. The script ensured that the selected SNPs maintained the proportion of SNPs per chromosome, resulting in a representative sample with a uniform distribution across all chromosomes (**Table S3**). STRUCTURE Harvester showed a delta K peak at K equal to 5, indicating that the panel consisting of 250 soybean genotypes can be divided into two subpopulations. Genome-wide association analysis was performed using the MLM and GLM in TASSEL (version 5.2.15) (Li et al., 2020). A significance threshold of LOD≈-Log(p) > 5.0 was used for each SNP. Kinship values were estimated using TASSEL (version 5.2.15) (Yu et al., 2006; Bradbury et al., 2007). The R software CMplot package were used to create Manhattan and QQ plots (Li et al., 2021).Three hundred SNP-based haplotype blocks were used in this GWAS. Haplotype blocks were constructed using the solid spline method implemented in Haploview 4.2 software (Barrett et al., 2005). The employed method assumes that the first and last markers within a block exhibit strong linkage disequilibrium (LD) with all intervening markers, thus offering more reliable block boundaries. A 1% cut off was applied, which implies that a SNP would not be incorporated into the block if its addition led to a recombinant allele frequency surpassing 1%. SNP markers significantly associated with seed coat color and situated within the same haplotype blocks were considered as potential regions for putative loci governing the traits under investigation.

### Metabolic pathway analysis

Based on gene function annotation in the Phytozome (https://phytozome.org/) and SoyBase (https://www.Soybase.Org) databases, candidate genes for soybean seed coat features were predicted. used metabolic pathway enrichment analysis from the Kyoto Encyclopedia of Genes and Genomes (http://www.kegg.jp/kegg/kaas/). The algorithm was used to determine the differences between groups that were statistically significant.


$$p=1-\sum_{1=0}^{m=1}\frac{(\frac{M}{i}(\frac{N-M}{N-i)}}{\left(\frac{N}{n}\right)}$$

Let N represent the total number of genes with KEGG annotation, while n denotes the number of candidate genes within N. Furthermore, M stands for the total number of genes annotated to specific pathways, and m signifies the number of candidate genes included in M.

### RNA isolation and qRT−PCR

Soybean grains exhibiting yellow and brown coat colors, which were significantly different and derived from parent plants in the RIL population, were collected during the R6 stage. The grains were immediately frozen in liquid nitrogen and stored at -80°C until RNA extraction. Total RNA was isolated from frozen samples using an RNeasy Plant Mini Kit (OMEGA, USA) and on-column DNase digestion (OMEGA, USA) following the manufacturer’s instructions. The RNA samples from yellow and brown coat colors were divided into two RNA pools (yellow_pool and brown_pool). Subsequently, 1,000 ng of RNA and TransScript One-Step gDNA Removal and cDNA Synthesis SuperMix (Vazyme, Nanjing, China) were employed for RNA extraction.

To validate the expression levels of candidate genes, quantitative real-time PCR (qRT-PCR) assays were conducted using gene-specific primers. The qRT-PCR reactions were performed in a 20 µL total volume containing 0.4 µL of each forward and reverse primer, 10 µL PerfectStart Green qPCR Master (TransGen, Beijing, China), 1.0 µL template (200-fold diluted cDNA), and 5.2 µL sterile water, using a Bio-Rad CFX96™ System (Bio-Rad Laboratories, CA, USA). The qRT-PCR program involved an initial 30 s at 95°C, followed by 39 cycles of 5 s at 95°C, 15 s at 60°C, and 12 s at 72°C, and a final extension of 5 s at 72°C. Soybean β-actin gene expression was used as an internal reference. The fold change in gene expression was calculated using the 2^(-ΔΔCt) method (Livak and Schmittgen, 2001). Each qRT-PCR reaction included three technical replicates.

## Results

### Phenotypic evaluation of the RILs and the natural population

**Figure 1:**
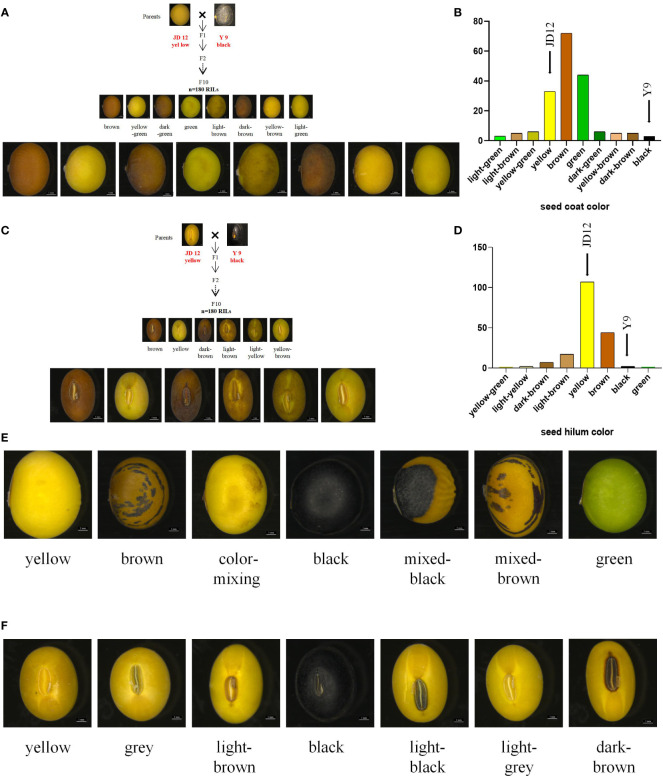
Phenotypic identification of RIL populations and natural population. Statistics the distribution of seed coat color and hilum color in two populations. **(A)** Distribution of seed coat color in RILs; **(B)** Column chart of the distrubution of seed coat color in RILs; **(C)** Seed hilum color distribution in RIL; **(D)** Column chart of the distribution of seed hilum color in RILs; **(E)** Color of seed coat distribution of in natural population; **(F)** Seed hilum color distribution in natural population. Scale bar, 1 mm.

Great phenotypic variation existed among the 180 RILs and a natural population material composed of 250 accessions. The RIL population, derived from the cross between cultivated soybean JD12 (yellow coat color, yellow hilum color) and wild soybean Y9 (black coat color, black hilum color), comprises 180 lines displaying a diverse array of seed coat colors. Brown was the most prevalent seed coat color, accounting for 39.56% of the population. Green (24.18%) and yellow (18.13%) followed in prevalence, with other less frequent colors such as yellow-green (3.30%), dark green (3.30%), yellow-brown (2.75%), light brown (2.75%), and dark brown (2.75%) also observed. The least common seed coat colors, black and light green, each constituted 1.64% of the population (**Figures 1A, B**; **Supplementary Table S1**). In terms of hilum color, the RIL population displayed a variety of hues, with yellow being the most common, representing 58.70% of the population. Other colors observed included light yellow (1.10%), light brown (9.34%), brown (24.16%), dark brown (3.85%), and black (1.67%). The least frequent hilum colors were yellow-green and green, each accounting for 0.55% of the population (**Figures 1C, D**; **Supplementary Table S2**). In the analysis of 250 accessions, the seed coat color distribution revealed that yellow was the most common color, representing 81.60% of the population. Other colors observed included green (8.40%), mixed-brown (2.00%), brown (1.60%), color-mixed (0.80%), and mixed-black, which was the least common at 0.40% (**Figure 1E**; **Supplementary Table S1**). Regarding hilum color, black was the predominant color, accounting for 32.00% of the population. Other hilum colors observed were yellow (14.40%), light brown (16.00%), brown (29.60%), dark brown (2.00%), grey (2.80%), light grey (2.40%), and light black, which was the least common at 0.80% (**Figure 1F**; **Supplementary Table S2**).

In the F_10_ RILs, representing a highly advanced generation population, the seed coat color and seed hilum color traits are qualitative and exhibit consistent phenotypes across different years. There are notable differences in seed coat color and seed hilum color among distinct lines. The 250 accessions comprise a collection of approved cultivated varieties and local varieties. The seed coat color and seed hilum color traits are qualitative in nature, displaying consistent phenotypes across various years, with no segregation observed over a three-year period. Nevertheless, considerable differences in seed coat color and seed hilum color can be found among different varieties (**Supplementary Table S3**). This study demonstrates significant phenotypic variation in seed coat and hilum colors among the 180 RILs and a natural population of 250 accessions. The F_10_ RIL population, derived from a cross between cultivated soybean JD12 and wild soybean Y9, shows consistent phenotypes for these traits across different years. A wide range of seed coat and hilum colors were observed in both populations.

### Construction of genetic linkage maps and Identification of QTLs

**Figure 2:**
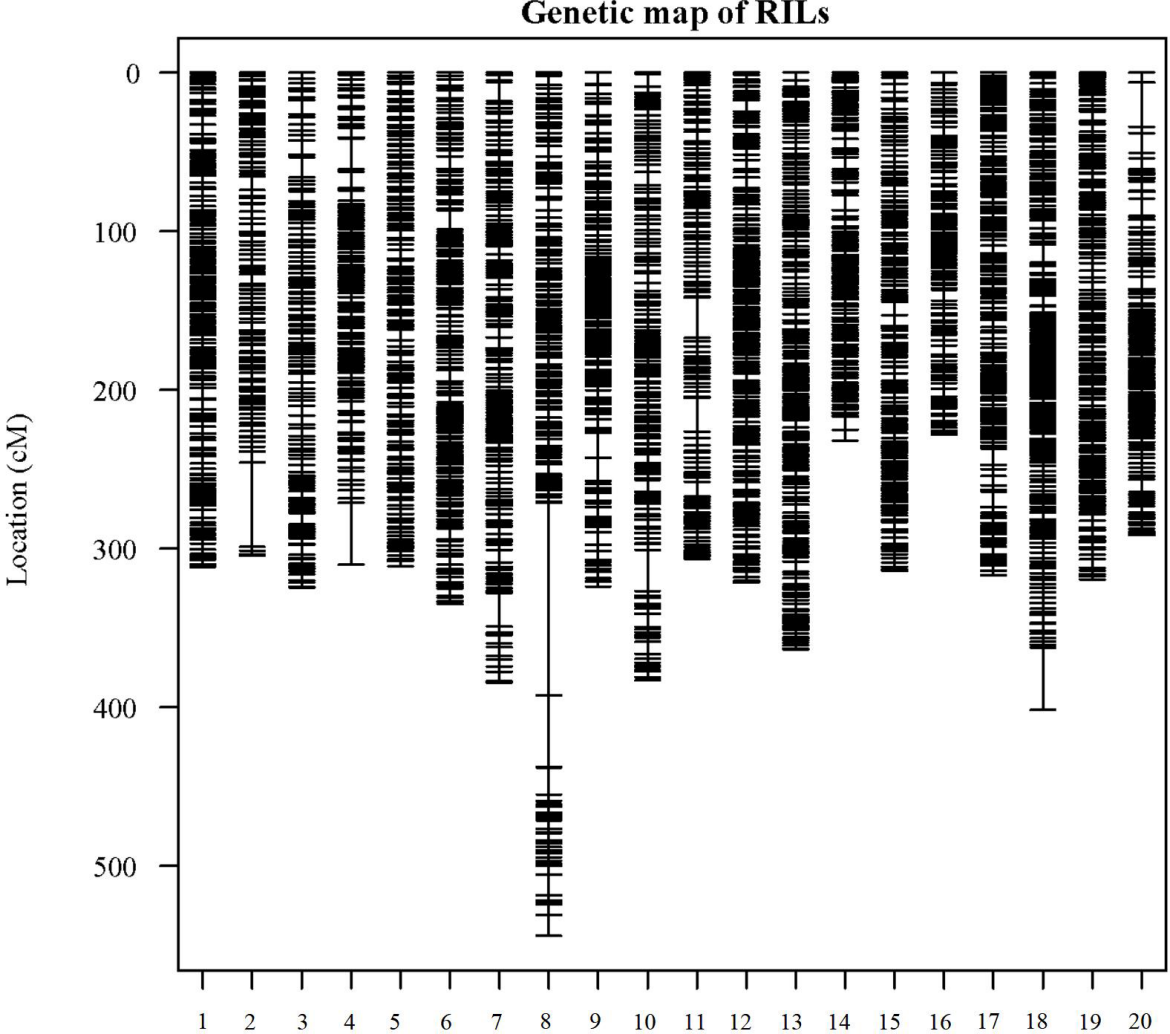
Distribution of genetic linkage maps on chromosomes. Genetic linkage map of RILs. Bin markers are distributed on 20 chromosomes. The black bars in each linkage group represent the mapped bin markers. The linkage group number is shown on the x-axis and genetic distance is shown on the y-axis (cM is the unit).

In the analysis of GBS sequences obtained from the 180 RIL population, a total of 6,288 high-quality SNPs were identified. Subsequently, the genotypic differences between the parental lines were examined, and sites with identical genotypes were excluded, resulting in the selection of 3,659 SNPs. The IciMapping software (Version 4.2.53) was employed to screen the SNP markers, yielding 1,732 bin markers. Utilizing one representative marker from each bin, a linkage map encompassing a cumulative length of 6,626.06 cM was constructed for this population. The number of markers on each linkage group ranged from 255 (chromosome 18) to 123 (chromosome 02), with an average of 183 markers per group. The extent of linkage distances covered by these markers varied between 543.96 cM (chromosome 08) and 228.16 cM (chromosome 16), averaging 129.6 cM. The mean inter-marker distances on the 20 chromosomes spanned from 1.41 to 2.71 cM, with an overall average distance of 1.81 cM (**Figure 2**; **Supplementary Table S4**).

In this study, we employed three distinct approaches, namely SMA, ICIM, and IM within the IciMapping software (Version 4.2.53), to discern significant QTLs linked with seed coat color and seed hilum color. A collective sum of 13 QTLs was ascertained to be associated with seed coat color and seed hilum color, surpassing the significance threshold of LOD ≥ 2.5. Among these QTLs, eight were correlated with seed coat color, located on chromosomes 2, 8, 9, 13, 15, 18, and 20. The proportion of phenotypic variance elucidated (PVE) by these loci ranged from 0.98% to 32.34%. Notably, *qSCC02* and *qSCC08* were detected by all three analytical methods, emphasizing their robustness in uncovering the genetic architecture underlying these traits. For *qSCC02*, the LOD scores obtained were 2.81, 3.81, and 3.81, while the corresponding proportions of PVE were 4.70%, 1.49%, and 1.49% for ICIM, IM, and SMA, respectively. In the case of *qSCC08*, the LOD scores were 16.08, 25.27, and 23.99, and the PVE were 32.34%, 7.78%, and 7.78% for ICIM, IM, and SMA, respectively. These results indicate the presence of significant associations between these QTLs and the traits of interest.

Five QTLs associated with seed hilum color were identified, located on chromosomes 02, 08, 14, 16, and 19 (**Table 1**). Five QTLs associated with seed hilum color were identified, located on chromosomes 02, 08, 14, 16, and 19 (**Table 1**). For *qSHC08*, congruent LOD scores of 5.66 were determined *via* both IM and SMA approaches, accompanied by identical proportions of PVE at 1.28% for each method. *qSHC14*, LOD scores of 2.85, 3.31, and 3.31 were achieved employing ICIM, IM, and SMA techniques, respectively. The PVE were found to be 6.16% for ICIM and 0.76% for both IM and SMA methods. Notably, *qSHC08*, which was detected using only two methods (IM and SMA), was situated within the same chromosomal interval as *qSCC08* (**Table 2**).

**Table 1:** QTL in RILs of soybean seed coat color and seed hilum color detected by different methods (ICIM, IM, SMA).

Trait	QTL Name	Position	Chr	Marker Interval	LOD	PVE(%)	Add	Method
Seed coat color	*qSCC02*	16	2	Chr02-2149517-Chr02-2418026	2.81/3.81/3.81	4.70/1.49/1.49	-0.58	ICIM/IM/SMA
	*qSCC08*	106	8	Chr08-8508938-Chr08-8679327	16.08/25.27/23.99	32.34/7.78/7.78	-0.47	ICIM/IM/SMA
	*qSCC08-1*	108	8	Chr08-8679152-Chr08-9054795	24.00	7.20	-2.19	IM/SMA
	*qSCC09*	124	9	Chr09-15059933-Chr09-10379226	3.14	1.21	-0.89	IM/SMA
	*qSCC13*	296	13	Chr13-18654598-Chr13-18585206	3.91	1.53	-1	IM/SMA
	*qSCC15*	262	15	Chr15-44574843-Chr15-44540308	5.04	8.28	-0.74	IM/SMA
	*qSCC18*	148	18	Chr18-44884534-Chr18-41231235	3.11	1.23	-0.89	IM/SMA
	*qSCC20*	283	20	Chr20-529767-Chr20-423389	2.53	0.98	-0.8	IM/SMA
Seed hilum color	*qSHC02*	74	2	Chr02-6147696-Chr02-7185126	3	0.58	-0.9	IM/SMA
	*qSHC08*	105	8	Chr08-8285888-Chr08-8508938	5.66	1.28	-1.35	IM/SMA
	*qSHC14*	42	14	Chr14-3839077-Chr14-4944933	2.85/3.31/3.31	6.16/0.76/0.76	-0.79	ICIM/IM/SMA
	*qSHC16*	78	16	Chr16-29250203-Chr16-29035407	3.28	0.77	-1.04	IM/SMA
	*qSHC19*	181	19	Chr19-37647577-Chr19-37737730	3.15	6.96	-0.89	ICIM

**Table 2:** QTL in RILs of soybean seed coat color and seed hilum color detected by three methods.

Trait	QTL Name	Chr	Position	Marker Interval	LOD	PVE(%)	Add	Method
Seed Coat Color	*qSCC02*	2	16	Chr02-2149517-Chr02-2418026	2.81/3.81/3.81	4.70/1.49/1.49	-0.583090379	ICIM/IM/SMA
	*qSCC08*	8	106	Chr08-8508938-Chr08-8679327	16.08/25.27/25.27	32.34/7.78/7.78	-0.47	ICIM/IM/SMA
Seed Hilum Color	*qSHC14*	14	42	Chr14-3839077-Chr14-4944933	2.85/3.31/3.31	6.16/0.76/0.76	-0.791780564	ICIM/IM/SMA

### Detection of seed coat and hilum color loci by GWAS

**Figure 3:**
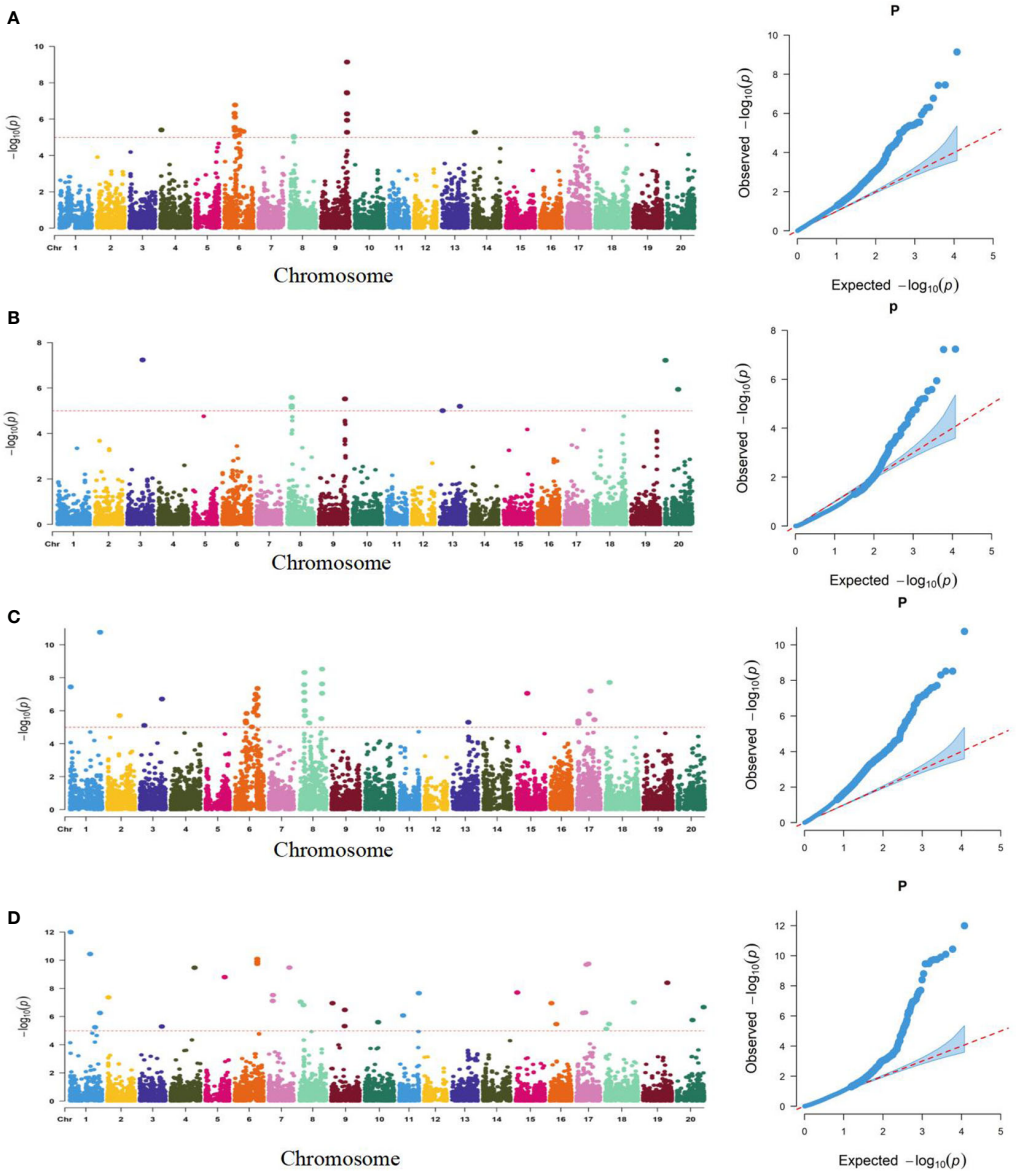
Manhattan (left) and Quantile-Quantile (Q-Q) Plots (right) of the Genome-Wide Association Study (GWAS) for Seed Coat Color and Seed Hilum Color Based on SNP Linkage Disequilibrium Blocks (SNPLDBs) in the Natural Population. **(A)**: Analysis of seed hilum color by GLM model **(B)**: Analysis of seed hilum color by MIM model **(C)**: Analysis of seed coat color by GLM model **(D)**: Analysis of seed coat color by MLM model. The dashed horizontal line represents the genome-wise significance threshold of 5. where the (-log P) values of the range from 1.1 12.

**Figure 4:**
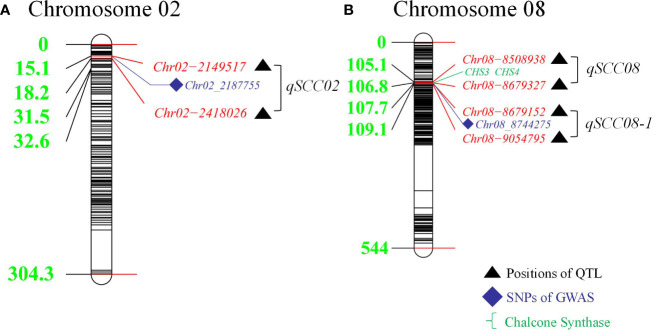
Distribution of Detected Quantitative Trait Loci (QTLs) in Linkage Groups on Chromosomes 2 and 8: Comparison of Results from Recombinant Inbred Lines (RILs) and Genome-Wide Association Study (GWAS) Analysis. **(A)** Distances (cM) of markers from the top of the linkage group. illustrating the colocalized loci of QTL and GWAS on chromosome 02. **(B)** Distances (cM) of markers from the top of the linkage group, presenting the colocalized loci of QTL and GWAS on chromosome 08.

Using a genetically diverse natural population consisting of 250 soybean accessions from both domestic and international sources, STRUCTURE software was employed to perform population structure analysis of 5129 informative SNPs, resulting in the identification of 2 subgroups (Ravelombola et al., 2021). TASSEL software was then utilized to identify significant quantitative trait nucleotides (QTNs) associated with seed coat color and seed hilum color using both GLM and MLM, and Manhattan plots and QQ plots were generated (**Figure 3**). A total of 21 and 16 SNPs were found to be associated with seed coat color and seed hilum color, respectively, at the significance level of LOD = 5 (**Supplementary Table S5**). In the natural population, the GLM method identified an seed coat color locus located at 8,744,275 bp on chromosome 8 that overlapped with the region containing *qSCC08_1* that was previously identified in the RILs. In addition, the MLM method detected an seed coat color locus at 2,187,755 bp on chromosome 02 that overlapped with the region containing *qSCC02* that was identified in the RILs (**Table 3**; **Figure 4**). The SNP located at 8,508,938 bp of the seed hilim color locus on chromosome 08 overlapped with the region including *qSHC08* that was previously identified in the RILs. Coincidentally, *qSCC08* was located in the same chromosomal interval as *qSHC08*.

**Table 3:** Multiple methods to locate segments in RILs and natural population.

Trait	QTL name	population	Chr	Position	LOD	PVE%	Method
Seed coat color	*qSCC02*	RILs	2	Chr02-2149517-Chr02-2418026	2.81/3.81/3.81	4.70/1.49/1.49	ICIM/IM/SMA
	Chr02-2187755	Natural population	2	Chr02-2187755	7.36	0.02/0.16	GLM/MLM
	Chr08-8744275	Natural population	8	Chr08-8744275	8.31	0.10	GLM
	*qSCC08_1*	RILs	8	Chr08-8679152-Chr08-9054795	24.00	7.20	IM/SMA
	*qSCC08*	RILs	8	Chr08-8508938-Chr08-8679327	16.08/25.27/25.27	32.34/7.78/7.78	ICIM/IM/SMA
	Chr08-8556756	Natural population	8	Chr08-8556756	7.58/5.21	0.12/0.08	GLM/MLM
Seed hilum color	Chr08-8508938	Natural population	8	Chr08-8508938	5.05/5.15	0.03/0.04	GLM/MLM
	*qSHC08*	RILs	8	Chr08-8508938-Chr08-8679327	16.08	1.28	ICIM/IM/SMA

**Figure 5:**
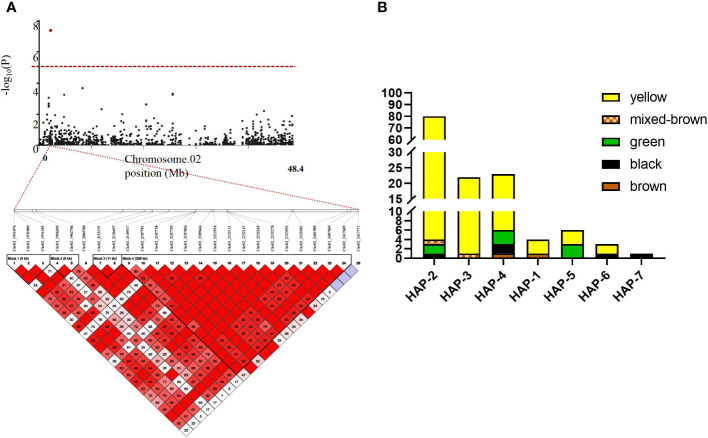
Linkage Disequilibrium (LD) Block Analysis of Major Quantitative Trait Nucleotides (QTNs) for Seed Coat Color on Chromosome 2 and Haplotype Analysis of Seed Coat Color Phenotypes in the Natural Population. **(A)** Hoplotype plot of Chr.02 **(B)** Haplotype Analysis of Seed Coat Color Phenotypes in the Natural Population. The x-axis represents the different haplotypes. while the y-axis shows the number of individuals with each seed coat color phenotype within each haplotype. Different colors in the bar char represent various seed coat colors.

In this study, we focused on the *qSCC02* region located on chromosome 02, which has been consistently associated with seed coat color variation in soybean. We performed a candidate gene prediction analysis in the 500 kb genomic region surrounding the peak SNP marker Chr.02_2187755. This analysis aimed to identify potential candidate genes responsible for seed coat color variation, providing valuable insights for future functional validation and molecular breeding endeavors. We conducted a haplotype block analysis using Haploview 4.2 software on the 500 kb candidate region, revealing a 268 kb genomic interval (block 4) containing 17 SNPs (**Figure 5A**), including the peak SNP marker Chr.02_2187755.

Subsequent detailed analysis revealed seven distinct haplotypes associated with seed coat color in this genomic interval. Such insights hold significant importance for the progression of plant genetics and breeding research. Haplotypes (Hap) 1-4 were predominantly associated with yellow seed coats, while Hap-5 showed a balanced association with half green and half yellow seed coats color. Notably, Hap-7 was exclusively linked to black seed coats (**Figure 5B**). This comprehensive understanding of the relationships between specific haplotypes and seed coat colors contributes to the advancement of plant genetics and breeding research, deepening our knowledge of the genetic basis for seed coat color variation in soybean.

### Candidate genes prediction and validation of stable QTLs

**Figure 6:**
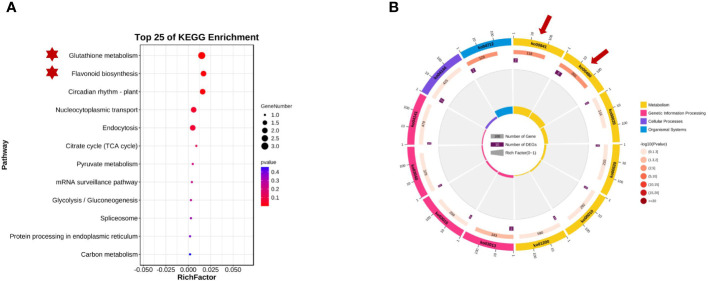
Kyoto Encyclopedia of Genes and Genomes (KEGG) enrichment analysisof qSCC028&qSHCO8. **(A)** Candidate Genes Enriched in Glutathione S-transferase (GST) Metabolic Pathway and Flavonoid Metabolic Pathway. **(B)** Enrichment of Candidate Genes in Metabolic Pathways on Chromosomes 2 and 8.

The KEGG database is the main public database for pathway analysis and can be used to systematically analyze the metabolic pathways and functions of gene products in cells. Referring to the KEGG database, 16 genes on chromosomes 2 and 8 were enriched in 12 specific metabolic pathway branches. Based on the KEGG analysis results (as shown in **Figure 6**), these data can help us quickly identify and predict metabolic pathways that may be related to plant seed coat color within the targeted regions, including glutathione metabolism (GST), flavonoid biosynthesis, circadian rhythm-plant hormone signal transduction, citrate cycle (TCA cycle), the mRNA surveillance pathway, glycolysis/gluconeogenesis, and carbon metabolism pathways.

*Glyma.08G110700* and *Glyma.08G110900* on chromosome 08 may be involved in soybean seed coat color. They encode chalcone synthases (CHSs: *CHS4A* and *CHS3C*) (in **Figure 4**) and participate in the biosynthesis of flavonoids. CHSs are key enzymes in the phenylpropanoid pathway branch that leads to the biosynthesis of flavonoid pigments, including anthocyanins. *Glyma.02G024600*, *Glyma.02G024700*, and *Glyma.02G024800* on chromosome 2 were enriched in the GST pathway, and previous research has shown that GST genes in plants are related to the transport and accumulation of anthocyanins. These five candidate genes all affected the composition and content of seed coat pigments in different ways. **Figure 4** shows the distribution of the detected QTLs among the linkage group.

**Figure 7:**
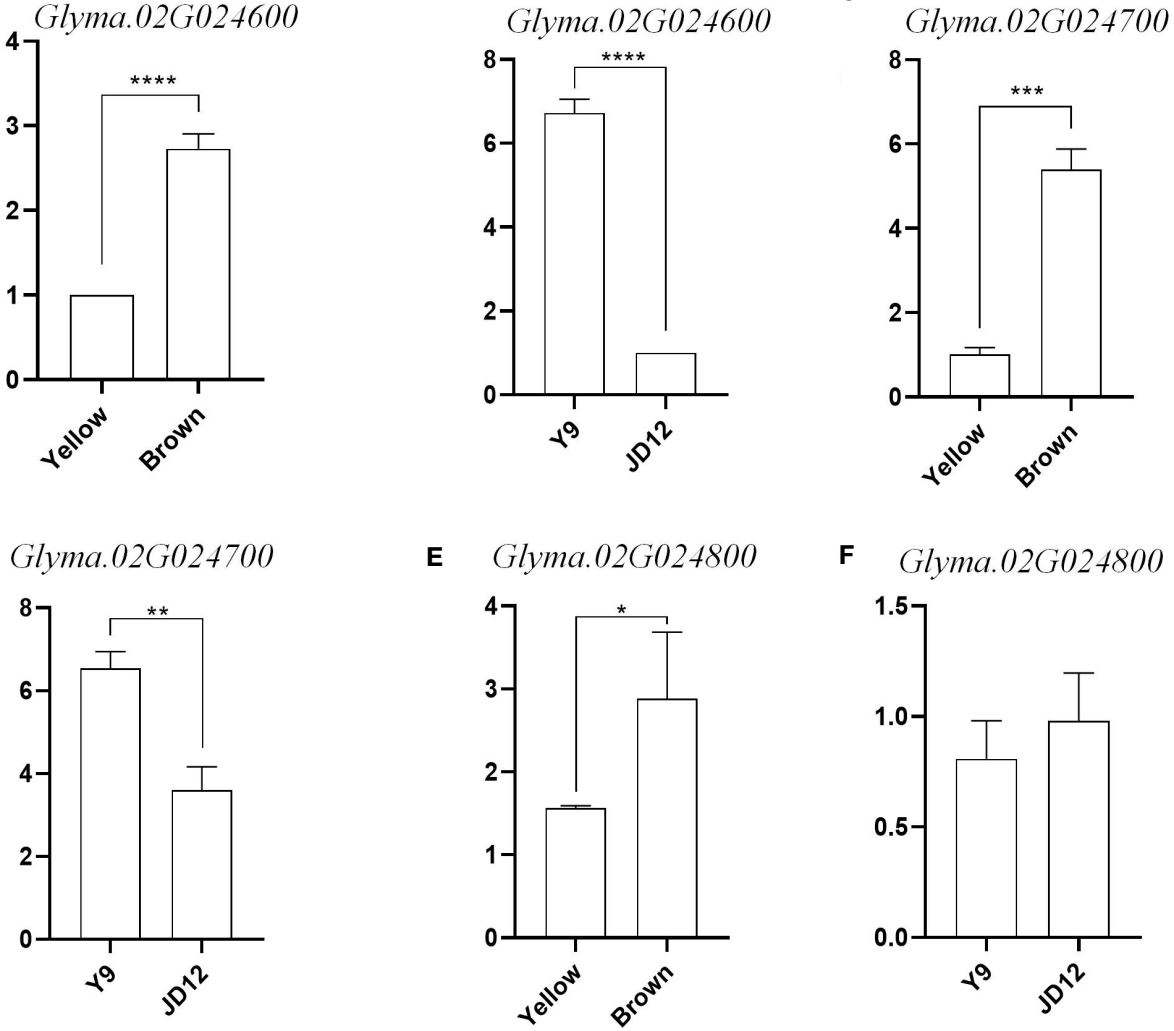
Relative expression levels of three candidate genes in the soybean grains of R6. **(A)** Relative expression of Glyma 02G024600 in yellow coat color and brown coat color; **(B)** Relative expression of Glyma.02G024600 in Y9 and JD12; **(C)** Relative expression of Gilyma. 02602: yellow coat color and brown coat color: **(D)** Relative expression of Glyma.02G024700 in Y9 and IDI12; **(E)** Relative expression of Glyma.02G024500 in yellow coat color and brown coat color; **(F)** Relative expression of Gilyma.02G024500 in Y9 and JD12. Sequence differences of the four candidate genes among the three parental genotypes and their transcript levels at different developmental stages of JDI7.*indicates difference at p< 0.05.**indicates significant difference at p< 0.01, ***significant at p< 0.001. and ****significant a p<0.0001.

To further determine whether candidate genes play a role in seed coat color and seed hilum color, we divided the grains with yellow coat color and brown coat color into two RNA pools (yellow_pool and brown_pool), and compared the expression of *Glyma.02G024600*, *Glyma.02G024700* and *Glyma.02G024800* in yellow_pool, brown_pool, JD12 and Y9. The results showed that the expression of *Glyma.02G024600* and *Glyma.02G024700* was significantly upregulated in Y9 (black coat color) and brown_pool at R6 compared with JD12 and yellow_pool. And the expression of *Glyma.02G024600* and *Glyma.02G024700* is nearly 3 times more in brown_pool than in yellow_pool. *Glyma.02G024600* and *Glyma.02G024700* are expressed in Y9 seven times and twice as much as JD12, respectively. *Glyma.02G024800* was different between the yellow_pool and brown_pool, but hardly not significantly different between parents (**Figure 7**). *Glyma.02G024600* and *Glyma.02G024700* both belongs to the tau subfamily. Combined with the data from gene annotation analysis, we believe that *Glyma.02G024600*, *Glyma.02G024700* and *Glyma.02G024800* were candidate genes for seed coat color in soybean.

## Discussion

### Enhancing QTL mapping precision by combining linkage and association analyses for seed coat color in soybean

With the rise and development of molecular biology techniques, marker-assisted selection (MAS) has been used to accelerate the breeding process (Li et al., 2020). Currently, QTL mapping and GWASs are commonly used to detect trait-associated regions and identify candidate genes (Torkamaneh et al., 2020). The importance of integrating linkage and association analyses for QTL mapping lies in the ability to utilize the complementary strengths of these two methods. Previous researchers have constructed linkage maps and physical maps using different types of genetic markers and have identified a large number of QTLs associated with agronomic traits in soybean using these markers (Song et al., 2004; Chan et al., 2012). Linkage analysis, based on the cosegregation of genetic regions in parental genomes, can allow more accurate mapping and uncover a wider range of genetic variation (Karikari et al., 2019). However, the construction of RIL populations is very challenging, and such populations can reveal only allelic diversity between the parents, with a resolution largely dependent on the number of recombination markers. Considering that QTL intervals usually extend over several centimorgans (cM), the limited number of molecular markers and uneven distribution of markers have limited the efficiency and accuracy of QTL mapping (Cregan et al., 1999; Marek et al., 2001; Li et al., 2017; Yu et al., 2020). In contrast, association analysis can identify QTLs with smaller effects and higher resolution. The study population for GWASs are natural populations composed of multiple types and breeds (Sonah et al., 2015), which can be used to detect loci in a genome that have multiple alleles. The GWAS approach has been used to identify target genes associated with traits in multiple species (Chang et al., 2018; Miao et al., 2020; Yan et al., 2021). However, this technique may have reduced power due to confounding factors such as population structure or unobserved environmental effects (Korir et al., 2013; Cao et al., 2017). Wang et al. (2022) used a population of RILs and a natural population composed of 455 accessions to jointly locate 26 height-related loci using linkage and association analysis. Similarly, *GmMPK1*, which encodes a mitogen-activated protein kinase, was identified as one of the candidate genes responsible for flavonoid content by the combined use of linkage analysis and association analysis (Wu et al., 2020). Shi et al. (2022) located candidate regions associated with salt stress using linkage and association analysis and identified *GsAKR1* as a candidate gene in this critical region based on tissue and induced expression analysis. Therefore, when combining these two methods, the strengths of each can compensate for the limitations of the other, resulting in more accurate and precise QTL mapping. In our study, we used a population of 180 RILs constructed by crossing cultivated soybeans with wild soybeans, as well as a natural population of 250 accessions from domestic and foreign germplasms. By using combined linkage and association analyses, we identified loci associated with seed coat color and seed hilum color. We consistently detected associations in the region from 8,508,938 bp to 8,679,327 bp on chromosome 8 with both methods. This region is located around the *I* locus and contains the gene cluster for CHS. This result indicates that our phenotypic assessments are accurate and validates the authenticity.

### Improving seed coat color localization accuracy by combining multiple models

In QTL mapping analysis, it is recommended to use multiple mapping methods and prioritize the QTLs that are commonly identified (Li et al., 2007; Su et al., 2010). Three mapping methods, SMA, ICIM, and IM, were used to detect a total of 13 QTLs associated with seed coat color and seed hilum color (**Table 3**). SMA, which considers only individual markers, identified a total of 12 QTLs. IM can utilize the positions of linked markers on chromosomes to systematically search for QTLs associated with the trait and can identify QTLs in supporting intervals. Using the IM model, we identified 12 QTLs. The effects of QTLs detected by the first two models may be absorbed by variables of the linked marker interval outside the marker interval, and the selection of different background markers can greatly affect the mapping results (Li et al., 2007; Li et al., 2008). Wang (2009) proposed the ICIM method, which first produces a linear model-corrected phenotype through stepwise regression and then locates additive-effect QTLs, avoiding the influence of background markers and improving QTL detection efficiency. Using this model, we detected 11 QTLs, of which three QTLs, *qSCC02*, *qSCC08* and *qSHC14*, were stably detected by all three methods.

In GWAS analysis, two GWAS models, the GLM and MLM, are used to improve the accuracy of the results. The GLM uses only Q population structure information, while the MLM uses Q + K. Due to its strict filtering requirements, the MLM may improve the accuracy of GWAS analysis. Previous studies have shown that compared to MLMs, GLMs have weaker stringency and accuracy (Huang et al., 2010; Yang et al., 2010). In addition, in practical research applications, it is recommended that multiple algorithm models be used for GWAS (Dhanapal and Crisosto, 2013; Zhang et al., 2017; Hickey et al., 2019) to reduce the limitations of false positives, complex population structure, and other factors. Combining linkage and association analysis, we detected two stable QTLs (*qSCC08* and *qSCC02*). The *qSCC08* QTL has been discovered to be situated at the well-established *I* locus, which governs seed coat color and is primarily controlled by the Inhibitor locus. As per Palmer et al. (2004), the *I* locus contains at least four classic genetic alleles, arranged in a hierarchy of dominance as follows: I (mainly colorless seeds) > *ii* (color confined to the hilum) > *ik* (“saddle;” color present at the hilum and slightly extending beyond it) > *i* (completely black seeds). The Inhibitor locus functions dominantly through a gain-of-function approach, displaying maternal-effect inheritance. As a result, seed coats exhibit a light yellow hue due to the absence of anthocyanins (Todd and Vodkin, 1993). The dominant Inhibitor (*I*) and *ii* alleles are attributed to natural gene-silencing effects that arise from connected yet distinct CHS gene clusters (chromosome 8, LG A2), which produce siRNAs targeting CHS gene transcripts exclusively in the seed coat for degradation (Clough et al., 2004; Senda, 2004; Tuteja, 2004; Kasai et al., 2007; Eckardt, 2009; Tuteja et al., 2009). In our study, we found *CHS3C* (*Glyma.08G110900*) and *CHS4A* (*Glyma.08G110700*), both belonging to the CHS gene family, to be potential genes within the *qSCC08* locus (Senda et al., 2002). These results indicate that the *qSCC08* locus has a clear effect on soybean seed coat color and demonstrate the accuracy and reliability of the results from this study. In addition, the other stably detected locus, *qSCC02*, was located on chromosome 02, which has not been reported in previous studies, and we believe it to be a new locus related to the regulation of seed coat color.

### Haplotype analysis and KEGG prediction of candidate genes

The association between genotype and phenotype is a fundamental concept in the field of genetics and is of paramount importance for plant breeding applications (Frankham, 1996; Bernardo, 2008). Haplotypes represent unique combinations of alleles or genetic variations located on a single chromosome. These combinations are inherited as a unit, facilitating the tracking of the inheritance patterns of particular traits within a population. Haplotypes have considerable utility in breeding applications, as they enable breeders to identify and select for desirable attributes in crop plants (Morrell et al., 2011). In our analysis, two distinct haplotypes (HAP-7 and HAP-2) are correlated with seed coat color phenotypes in a plant population. HAP-7 (GGCCCCTGTTTGCACT) is exclusively associated with the black seed coat phenotype, suggesting that plants possessing this haplotype exhibit a specific genetic variation that culminates in the formation of black seed coats. Conversely, HAP-2 (GACTTGTCCACGCACT) predominantly corresponds to the yellow seed coat phenotype, with 76 individuals within this haplotype group displaying yellow seed coats. The principal distinction between the haplotypes lies in their respective sequences: HAP-7 contains “GCCCCTGTTT” while HAP-2 encompasses “ACTTGTCCAC”. It is crucial to recognize that while the majority of plants harboring HAP-2 manifest yellow seed coats, some variation may still exist due to the influence of additional genes or environmental factors. Comprehending these haplotype variations and their corresponding phenotypes enables plant breeders to make judicious choices in selecting particular seed coat colors for their breeding objectives. By leveraging the genotype-phenotype relationships within haplotypes, breeders can enhance the efficiency and precision of their breeding programs, ultimately leading to the development of cultivars with desired characteristics (Hall et al., 2019).

Upon examining the 2,149,517 bp to 2,418,026 bp region where the stable *qSCC02* locus was identified, we discovered a total of 16 genes. KEGG enrichment analysis revealed that three of these genes were involved in the GST metabolic pathway. Glutathione S-transferases are known for their capacity to metabolize harmful compounds. In plants, GSTs play crucial roles in cellular metabolism and detoxification, and they have been widely investigated for their participation in herbicide and insecticide detoxification processes (Mannervik et al., 1988; Edwards et al., 2000; Liang et al., 2003). Moreover, plant *GSTs* also impact plant secondary metabolites like anthocyanins and cellular responses to auxins (Gao et al., 2020). The *GST* gene family has been analyzed in the whole genomes of plants such as Arabidopsis thaliana (Wagner et al., 2002) and maize (McGonigle et al., 2000). Anthocyanins are abundant, natural, water-soluble plant pigments, and their content is related to seed coat color (Caldas and Blair, 2009). Cyanidin and pelargonidin are the final products of flavonoid biosynthesis (Holton, 1995; Winkel-Shirley, 2001), and anthocyanins are synthesized in the endoplasmic reticulum and localized in vacuoles (Wagner et al., 2002). Research has shown that *GST* genes in plants are involved in anthocyanin transport; for example, the mutant gene Bronze-2, which is involved in anthocyanin transport in maize, causes the production of a bronze-colored pigment in cells, resulting in improper accumulation of anthocyanins (Mueller et al., 2000). Bz2 in maize plays an important role in the output of anthocyanins to vacuoles (Marrs et al., 1995). In Arabidopsis thaliana, *AtTT19* is required for anthocyanin transport (Kitamura et al., 2004). In addition to *ZmBz2*, these genes also belong to the phi subfamily, while *ZmBz2* belongs to the tau subfamily (Marrs et al., 1995; Larsen et al., 2003). Previous studies have shown that a decrease in GST function may lead to a reduction in anthocyanin accumulation.

Insertions and deletions (indels) in *GST* gene bases have been shown to cause white flowers in some plant species, including peach, apple, and others (Cheng et al., 2015; El-Sharkawy et al., 2015; Lu et al., 2021). *GST* genes encode glutathione S-transferases, which are enzymes involved in various cellular processes, including the detoxification of harmful substances, regulation of cellular redox status, and modulation of signaling pathways. In peach, the *PpGST1* gene has been found to regulate anthocyanin accumulation through interaction with the transcription factor *PpMYB10.1* (Zhao et al., 2020). Anthocyanins are pigments responsible for the red, blue, and purple colors in many plant tissues, including flowers, fruits, and leaves. They have a role in attracting pollinators, protecting against UV radiation, and acting as antioxidants. The R6 stage is a specific growth stage in plants, although its definition may vary among different plant species. In soybean, the R6 stage represents a critical phase during which flowers form pods, and seeds in the pods at the top node have filled the pod cavity (Boerma et al., 2004). This stage is essential for studying seed coat color and other biological characteristics, as seed growth and development are highly active. A study on soybean plants revealed that three *GST* genes (*Glyma.02G024600*, *Glyma.02G024700*, and *Glyma.02G024800*) may be involved in anthocyanin accumulation or transport. Further functional verification and marker development for these candidate genes can provide a foundation for exploring genes related to seed coat color regulation in soybeans and other crops. Understanding the genetic basis of anthocyanin accumulation and the role of *GST* genes in this process can contribute to plant breeding efforts aimed at improving crop quality, yield, and stress resistance.

## Data availability statement

The data presented in the study are accessible via this link: https://bigd.big.ac.cn/gvm/getProjectDetail?Project=GVM000541.

## Author contributions

JQ conceived and designed the experiments. YY, TZ, LL and FW performed the experiments. YY, TZ, and LL carried out the bioinformatics analysis. YY and TZ wrote the manuscript. KZ, BL, CY and YQ gave insightful suggestions. JQ improved the manuscript errors and English language. All authors contributed to the article and approved the submitted version.
